# Editorial: Metabolically Healthy and Unhealthy Obese Children and Adolescents

**DOI:** 10.3389/fendo.2020.613703

**Published:** 2020-10-28

**Authors:** Claudio Chiesa, Lucia Pacifico, Bo Xi, Cristina Cadenas-Sanchez

**Affiliations:** ^1^Institute of Translational Pharmacology, National Research Council (CNR), Rome, Italy; ^2^Department of Maternal and Child Health, Sapienza University of Rome, Rome, Italy; ^3^Department of Epidemiology, School of Public Health, Shandong University, Jinan, China; ^4^Institute for Innovation & Sustainable Development in Food Chain (IS-FOOD), Public University of Navarra, Pamplona, Spain

**Keywords:** metabolic phenotypes, obesity, children, organ damage, nonalcoholic fatty liver disease

## Introduction

It is well established that pediatric obesity is closely associated with an increased risk of metabolic diseases, all of which lead to marked increase in cardiovascular disease (CVD) events in adulthood (1). Therefore, there is a need for an early identification and treatment of youths who are at greater risk of developing obesity-related complications (2). A growing interest has been raised regarding a subgroup of obese individuals, including children, who are metabolically healthy, for whom the term “metabolically healthy obese (MHO)” has been proposed (3, 4). Since MHO in adults has its origin in the childhood years (5), stratification of obese youths, based on metabolic heath status, might be of crucial importance to improve in early ages prevention strategies for cardiometabolic diseases.

This Research Topic draws together seven original articles, and one comprehensive review (Vukovic et al.) summarizing the current knowledge on the definition, epidemiology, predictors, underlying mechanisms, and treatment outcomes of the MHO phenotype in the pediatric population.

## Criteria to Identify MHO and Epidemiology

In recent years, a voluminous literature has accumulated, advocating different definitions of MHO in children. However, the use of non-harmonized definitions of MHO has been a major problem, making it difficult to compare prevalence data among studies. As such, the MHO prevalence has been estimated to range between 7% and 21% in youths, notably between 3% and 87% in those with overweight/obesity (Vukovic et al.). In one international cohort including 3,497 children, 4.5% were classified as MHO according to the modified National Cholesterol Education Program (NCEP) criteria, while 8.2% according to the modified International Diabetes Federation criteria (6). Yet, in a national survey of 4,200 Iranian children, the prevalence of MHO was 10.4% according to the modified NCEP criteria (7). For these reasons, an international panel of 46 experts has recently convened to create consensus (≥80% agreement) on an evidence-based definition of MHO in children (8). The experts agreed on using the World Health Organization body mass index criteria to assess weight status, and including high-density lipoprotein cholesterol >40 mg/dl, triglycerides ≤150 mg/dl, systolic and diastolic blood pressure ≤90^th^ percentile, and a measure of glycemia to define MHO status.

## Risk of Target Organ Involvement

Structural and functional cardiovascular modifications including left ventricle hypertrophy, systolic/diastolic dysfunction, and increased carotid intima-media thickness have been considered preclinical indices of CVD in pediatric and adult obese subjects (9, 10). Nonalcoholic fatty liver disease (NAFLD) is increasing globally, and is currently the most common cause of chronic liver disease among youths worldwide (11). Two research manuscripts focused on organ damage such as cardiovascular complications and NAFLD in obese children. Corica et al. demonstrated a negative effect of childhood obesity on subclinical structural and functional cardiovascular modifications. Particularly, severity of overweight, abdominal obesity, and insulin resistance were the main predictors of cardiovascular remodeling, subclinical myocardial dysfunction, and amount of epicardial adipose tissue. Metabolically unhealthy obese (MUO) patients showed a significant unfavorable cardiometabolic profile compared to MHO subjects. Ting et al. demonstrated that liver steatosis was present in 77.2% of overweight/obese children, and that those with metabolic syndrome were more likely to have advanced liver fibrosis. Also, among children with NAFLD waist circumference predicted risk of liver fibrosis. Overall, these studies suggest that a distinction between MHO and MUO phenotypes might be useful in planning a personalized follow-up approach in obese youths.

## Determinants and Predictors of Metabolic Health Status

The distribution of adipose tissue has a critical role in the metabolic health status. There is an emerging evidence that ectopic adipose tissue deposition is a strong predictor of MUO phenotype (12). In addition to the articles by Corica et al. and Ting et al. pointing to abdominal fat accumulation as predictor of systemic metabolic changes, another point of interest is the article by Payab et al. who showed that in 4,200 children, hip, neck, and wrist circumferences were significantly associated with obesity phenotypes and their metabolic status. These measures may represent innovative, low-cost, and alternative tools for assessing obesity and metabolic syndrome in children. Genovesi et al. in a large pediatric population showed that non-traditional cardiovascular risk factors such as serum uric acid, homeostasis model assessment of insulin resistance index, and waist-height ratio values were independent predictors of being MUO, suggesting that the current definition of MHO may lead to an underestimation of the number of obese children actually at risk for early organ damage.

Hepatic fat content is also an important estimate of systemic metabolic health (12). The identification of potential biomarkers of NAFLD and metabolic syndrome is an important clinical agenda. The study by Mörwald et al. revealed that magnetic resonance imaging-assessed liver fat content and high-sensitivity C-reactive protein best predicted serum ferritin values. These results suggest that ferritin may serve as a marker of early fatty liver disease in childhood obesity, at least in males. This is of interest, as non-invasive surrogate scores such as the fatty liver index have been shown to poorly predict liver fat content in obese children.

There is another aspect which is worth mentioning: the inverse association of vitamin D with obesity, metabolic syndrome, and insulin resistance, in light of its numerous extra-skeletal roles and functions. Esmaili et al. found that vitamin D status and metabolic health display a significant interaction in children and adolescents. The prevalence of hypovitaminosis D was markedly higher among MUO, followed by metabolically unhealthy non-obese, and MHO groups.

## Role of Lifestyle Factors

Bluher and Schwarz first suggested that lifestyle factors, such as level of physical activity or cardiorespiratory fitness, may play a key role in determination of MHO phenotype (13). Accordingly, a recent systematic review and meta-analysis (14) showed that MHO individuals are more active, spend less time in sedentary behaviors, and have higher level of cardiorespiratory fitness than MUO subjects. In particular, among children and adolescents (14), higher levels of moderate-to-vigorous physical activity, higher cardiorespiratory fitness levels as well as a greater reduction in sedentary behaviors are present in MHO versus MUO children (15–18). Furthermore, in the present Research Topic, Mayerhofer et al. showed that multidisciplinary intervention including exercise, nutritional counseling, and psychotherapy significantly improved body composition, insulin resistance, and inflammation in obese youths.

## Conclusions

This Research Topic provides an important and timely update on metabolic phenotypes associated with obesity in youths. Future large prospective studies are needed to examine the transition between MHO and MUO, and the interplay between genetics and lifestyle factors in both development and reversal of such phenotypes.

## Author Contributions

CC, LP, BX, and CC-S, conceptualized, designed, wrote and approved the Editorial. All authors contributed to the article and approved the submitted version.

## Funding

This work was in part supported by National Natural Science Foundation of China (81722039,81673195) and by the Spanish Ministry of Science and Innovation (CC-S, FJC2018-037925-I).

## Conflict of Interest

The authors declare that the research was conducted in the absence of any commercial or financial relationships that could be construed as a potential conflict of interest.
